# A Rapid Method for Detection of *Salmonella* in Milk Based on Extraction of mRNA Using Magnetic Capture Probes and RT-qPCR

**DOI:** 10.3389/fmicb.2019.00770

**Published:** 2019-04-05

**Authors:** Yalong Bai, Yan Cui, Yujuan Suo, Chunlei Shi, Dapeng Wang, Xianming Shi

**Affiliations:** ^1^ MOST-USDA Joint Research Center for Food Safety, School of Agriculture and Biology and State Key Laboratory of Microbial Metabolism, Shanghai Jiao Tong University, Shanghai, China; ^2^ Institute for Agri-food Standards and Testing Technology, Shanghai Academy of Agricultural Sciences, Shanghai, China

**Keywords:** amino-modified silica-coated magnetic nanoparticles, magnetic capture probes, *Salmonella*, RT-qPCR, milk

## Abstract

Magnetic separation is an efficient method for target enrichment and elimination of inhibitors in the molecular detection systems for foodborne pathogens. In this study, we prepared magnetic capture probes by modifying oligonucleotides complementary to target sequences on the surface of amino-modified silica-coated magnetic nanoparticles and optimized the conditions and parameters of probe synthesis and hybridization. We innovatively put the complexes of magnetic capture probes and target sequences into qPCR without any need for denaturation and purification steps. This strategy can reduce manual steps and save time. We used the magnetic capture probes to separate *invA* mRNA from *Salmonella* in artificially contaminated milk samples. The detection sensitivity was 10^4^ CFU/ml, which could be increased to 10 CFU/ml after a 12 h enrichment step. The developed method is robust enough to detect live bacteria in a complex environmental matrix.

## Introduction

Magnetic nanoparticles and especially immunomagnetic nanoparticles have been widely used for foodborne pathogen detection (Escalante-Maldonado et al., 2015; Sun et al., 2015; Hwang et al., 2016; Luciani et al., 2016; Wang et al., 2016). The labeled antibody is a key point for a successful immunomagnetic detection method, and a limiting step is the quality of the anti-pathogen antibody used. The genus *Salmonella* is especially problematic because it has over 2,600 serotypes, so the probability of false negative may be high (Eng et al., 2015). In addition, the cost of superior anti-bacteria antibodies was always very high.

Developments in molecular biology, genomics, and bioinformatics now enable specific nucleotide sequences to be developed as barcodes for detection of target pathogens. Furthermore, the nucleotide sequence adjacent to the pathogen-specific barcode can also be used as a medium to purify the detection sequence. For example, magnetic nanoparticles labeled with complementary sequences were used to capture target DNA sequences containing barcodes of *Listeria monocytogenes* followed by amplification and identification by polymerase chain reaction (PCR) (Amagliani et al., 2004). They further used magnetic capture probes to simultaneously isolate *Salmonella* and *L. monocytogenes* DNA from seafood and detected the barcodes by triplex real-time PCR (Amagliani et al., 2010). However, since genomic DNA maintains a double helical structure, even when genomic DNA was denatured, the sensitivity would be compromised because of the large size of the genomic DNA.

Alternatively, the use of mRNA, which is single-stranded and much smaller than genomic DNA, for oligonucleotide hybridization may improve the procedure. The stability of DNA:RNA hybrids is also substantially greater than those of DNA:DNA duplexes (Casey and Davidson, 1977). Moreover, mRNA is considered as a more appropriate target than DNA to assess cell viability because mRNA have a short half-life, only a few minutes (Coutard et al., 2005; Liu et al., 2010), and is generally present only in viable cells (Garcia et al., 2010). That is, only viable cells could be detected using RNA-based detection methods (Zhou et al., 2014), which is the true harmful risk to food safety.

In previous research, we found nanoparticles affected PCR primarily *via* surface interactions with PCR components, and if the surface was blocked, the inhibition effect would be eliminated (Bai et al., 2015). Therefore, we proposed to directly add the complexes of magnetic capture probes and the captured target sequences in RT-qPCR to detect *Salmonella*, seeking to reduce operation steps and target losses, save time, and enhance sensitivity.

In this study, we prepared magnetic capture probes by modifying oligonucleotides complementary to target sequences on the surface of amino-modified silica-coated magnetic nanoparticles and optimized the conditions and parameters of probe synthesis and hybridization. We used the magnetic capture probes to separate *invA* mRNA, with the novel step of putting the complexes of magnetic capture probes and *invA* mRNA into RT-qPCR mixture without any denaturation and purification steps, to detect *Salmonella* in milk.

## Materials and Methods

### Reagents

Glutaraldehyde, 4-(N-Maleimidomethyl) cyclohexane-1-carboxylic acid 3-sulfo-N-hydroxysuccinimide ester sodium salt (SMCC), Triton X-100, lysozyme, and proteinase K were purchased from Sigma-Aldrich (St. Louis, MO, USA). 2 × SYBR Green PCR mix was obtained from TaKaRa (Dalian, China). All preparations and measurements were carried out in sterilized Millipore water. PCR primer pairs and oligonucleotides were synthesized by Shanghai Biotech (Shanghai, China). The detailed information was shown in Table 1. All the oligonucleotides and primer pairs were designed for this study except the primers pair InvA-f/r used for *invA* of *Salmonella* that have been previously described (Jiang et al., 2013).

**Table 1:** Oligonucleotides and primer pairs in this study.

Designation	DNA sequence (5′ to 3′)
**Capture oligonucleotides**	
Capture Oligonucleotides1	NH_2_-TTTTTTTTTTTTTTT ATTCCGCCGTGTATCGTAATTGAGT
Capture Oligonucleotides2	HS-TTTTTTTTTTTTTTTATTCCGCCGTGTATCGTAATTGAGT
Capture Oligonucleotides3	ACAGTACCGCAGGAAACGTTGTTTTTTTTTTTTTTT3′-SH
**Long oligonucleotides**	
Long Oligonucleotides1	GAAGAGATTTTAGCGCAGTGTAGCATTACTGGATACTGCGATTATTGAACTCAATTACGATACACGGCGGAAT
Long Oligonucleotides2	GAAGAGATTTTAGCGCAGTGTAGCATTACTGGATACTGCGATTATTGAACTCAATTACGATACACGGCGGAATTTTTTTTTTTTTTTT-SH
**Primer pairs**	
Lolig-f/r	GAAGAGATTTTAGCGCAGTGTAG; CATTACTGGATACTGCGATTATTGA
InvA-f/r	ACAGTGCTCGTTTACGACC; ACTGGTACTGATCGATAAT

### Strains and Cultivation

The Guangdong Institute of Microbiology (Guangdong, China) provided *Salmonella enterica* (ATCC 13076). *Salmonella* was cultivated using Luria-Bertani medium (Becton Dickinson, MD, USA). Milk was obtained from a local dairy and tested negative for *Salmonella* by selective plating and PCR methods before use.

### Synthesis and Analysis of Amino-Modified Silica-Coated Fe_3_O_4_ Magnetic Nanoparticles

Fe_3_O_4_ magnetic nanoparticles, which were prepared using the co-precipitation method (Bai et al., 2015), were coated with silica and modified with amino groups by the reverse microemulsion method (Bai et al., 2016). ASMNPs morphologies were observed and analyzed by transmission electron microscopy (TEM) using a JEM-2010HT instrument (JEOL, Japan). The particles were sonicated for 1 min, and 10 μl of the solution was placed on a 200 mesh copper grid and then dried at room temperature. The grid was used for TEM analysis.

### Preparation of Magnetic Capture Probes

**Figure 1:**
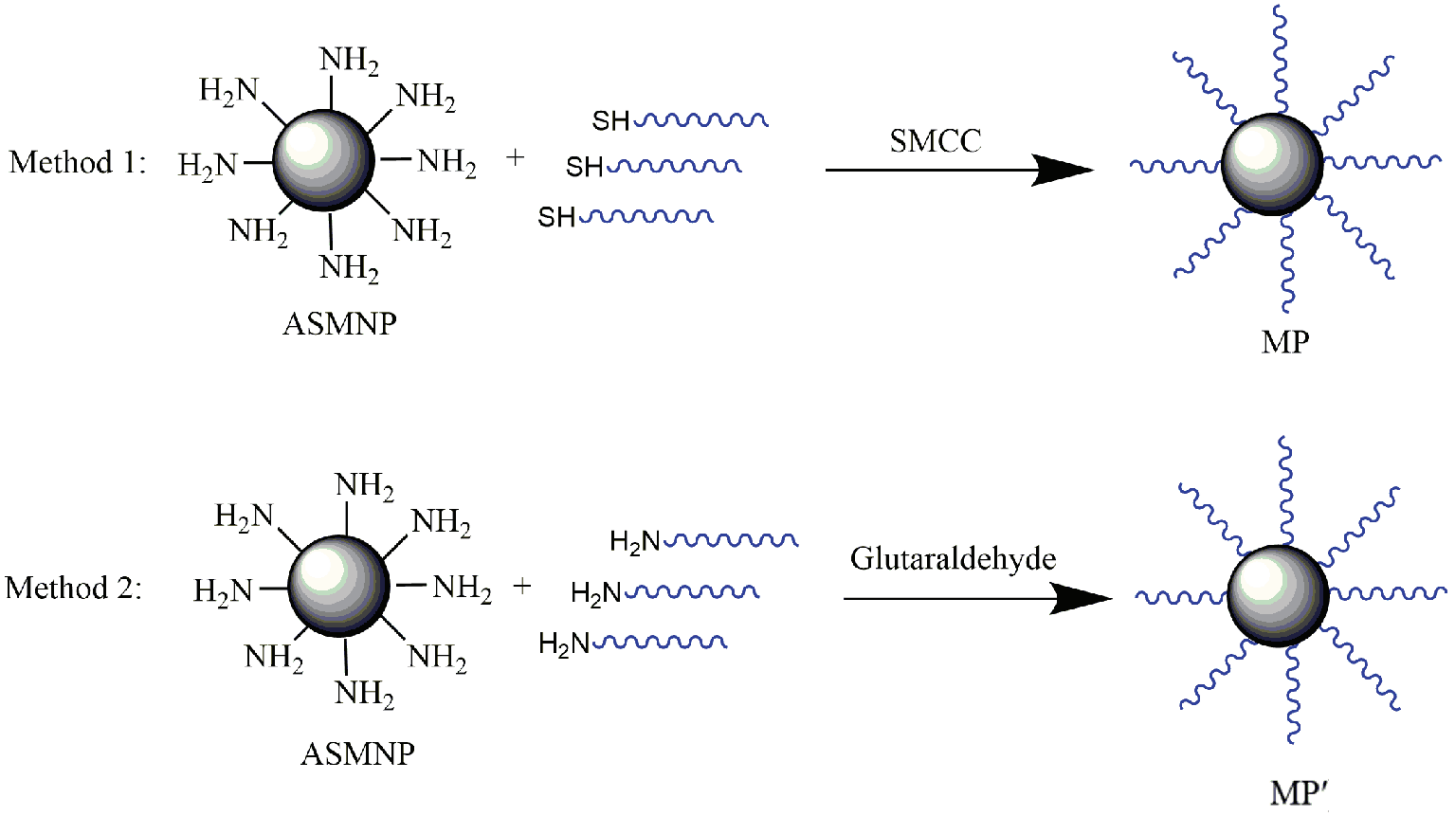
The schematic diagrams of preparation of SMCC-based MP and glutaraldehyde-based MP′.

Two methods were used to label capture oligonucleotides on the ASMNPs surfaces based on the previous reports (Nam et al., 2004; Bruce and Sen, 2005). The schematic diagrams were shown in Figure 1.

*Method 1*: ASMNPs (2 mg) were dispersed in 1 ml phosphate buffered saline (PBS) buffer containing 200 μg SMCC, pH 7.4. After shaking for 8 h, the pellets were dispersed in 1 ml of Capture Oligonucleotide2 solution (10 μmol/L) and shaken overnight at room temperature. The pellets were blocked with 10% skimmed milk powder solution for 8 h and dispersed in PBS buffer containing 2% BSA and 0.1% sodium azide. The final magnetic capture probe was named MP.

*Method 2*: ASMNPs (2 mg) were dispersed in 1 ml phosphate buffered saline (PBS) containing 5% glutaraldehyde (pH 7.4). The magnetic pellets were washed with PBS to remove free glutaraldehyde after shaking for 3 h, and the pellets were suspended in 500 μl PBS containing 200 μl Capture Oligonucleotide1 solution (10 μmol/L) and incubated overnight at room temperature. The magnetic pellets were blocked with 100 μl of 10% skimmed milk powder solution (Solarbio, Shanghai, China) and suspended in PBS containing 2% BSA and 0.1% sodium azide. The final magnetic capture probe was named MP′.

### Capture Using Magnetic Capture Probes

Samples were pretreated based on a modification method of published procedures (Amagliani et al., 2004; Bai et al., 2013). In brief, artificially contaminated milk samples (10 ml) were centrifuged at 6000 ×g for 20 min at 4°C, and the pellets were suspended in 1 ml of RNAprotect Bacteria Reagent (Qiagen, Germany). They were then incubated for 5 min and centrifuged at 9400 ×g for 10 min. The pellets were suspended in 50 μl of 50 mg/ml lysozyme, 50 μl of 20 mg/ml proteinase K, and 10 μl of Triton X-100 and incubated at 37°C for 15 min. Trizol (Invitrogen, Carlsbad, CA, USA) was added (1 ml) and the solution was incubated for 5 min at room temperature. Chloroform (250 μl) was added and the solution was vortexed for 15 s. After centrifugation at 9600 ×g for 10 min, the upper layer (aqueous phase) was transferred to a new 1.5 ml tube and incubated with 50 μg magnetic capture probes labeled with Capture Oligonucleotides3, which was partly complementary to *invA* mRNA, for 15 min at room temperature. The pellets were magnetically separated and mixed with 2.5 μl of 10X DNase buffer and 1 μl of DNase (Takara, Dalian, China) then incubated at 37°C for 20 min. The reaction was stopped by heating at 80°C for 2 min after the addition of 2.5 μl of 0.5 mol/L EDTA. The pellets were washed with DEPC-treated water and then used as templates in RT-qPCR.

### QPCR and RT-qPCR Amplification

Quantitative PCR (qPCR) was performed using 25 μl reaction volumes containing 1 μl of DNA template, 5 pmol of each primer, and 12.5 μl of 2× SYBR® Green PCR master mix (TaKaRa, Dalian, China). PCR thermocycling was as follows: 2 min at 95°C, 40 cycles of 15 s at 95°C, 15 s at 60°C, and 20 s at 72°C. Amplifications were carried out using a Mastercycler^®^ ep realplex instrument (Eppendorf, Germany). RT-qPCR was performed using a One Step PrimeScript RT-qPCR Kit (TaKaRa, Dalian, China) according to the manufacturer’s instructions.

## Results

### Optimization of Magnetic Capture Probes

**Figure 2:**
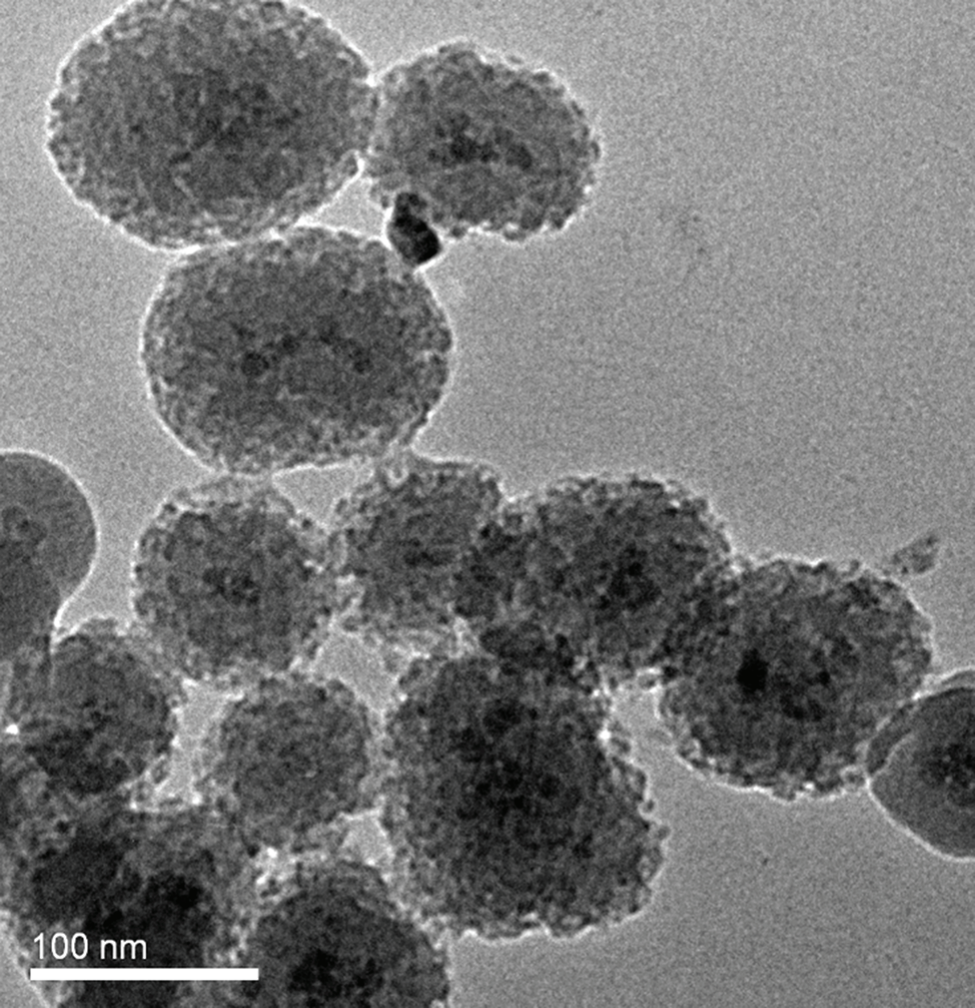
TEM image of ASMNPs synthesized for this study.

The spherical amino-modified silica-coated Fe_3_O_4_ magnetic nanoparticles we synthesized appeared rough (Figure 2), different to the particles with smooth surfaces in previous reports (Bai et al., 2016). In our previous study, we found that this rough surface may be the reason for the large number of surface amino groups available for coupling (Bai et al., 2016). Using magnetic nanoparticles with many more amino groups was a basic strategy to maximize the capture efficiency of probes. Additionally, binding more capture oligonucleotides to the amino groups is another critical step.

There are two basic strategies to label the oligonucleotides on ASMNPs. One strategy is to use SMCC as a coupling agent to covalently immobilize the thiol-modified oligonucleotides (Capture Oligonucleotides1) on the surface of ASMNPs (the final magnetic capture probe was named MP); another strategy is to use glutaraldehyde as a coupling agent to covalently immobilize the amino-modified oligonucleotides (Capture Oligonucleotides2) to ASMNPs (the final magnetic capture probe was named MP′). The schematic diagrams were shown in Figure 1. We used Long Oligonucleotides1 whose 3′ end was complementary to Capture Oligonucleotides1 as target to compare the capture efficiency of these two types of probes.

**Figure 3:**
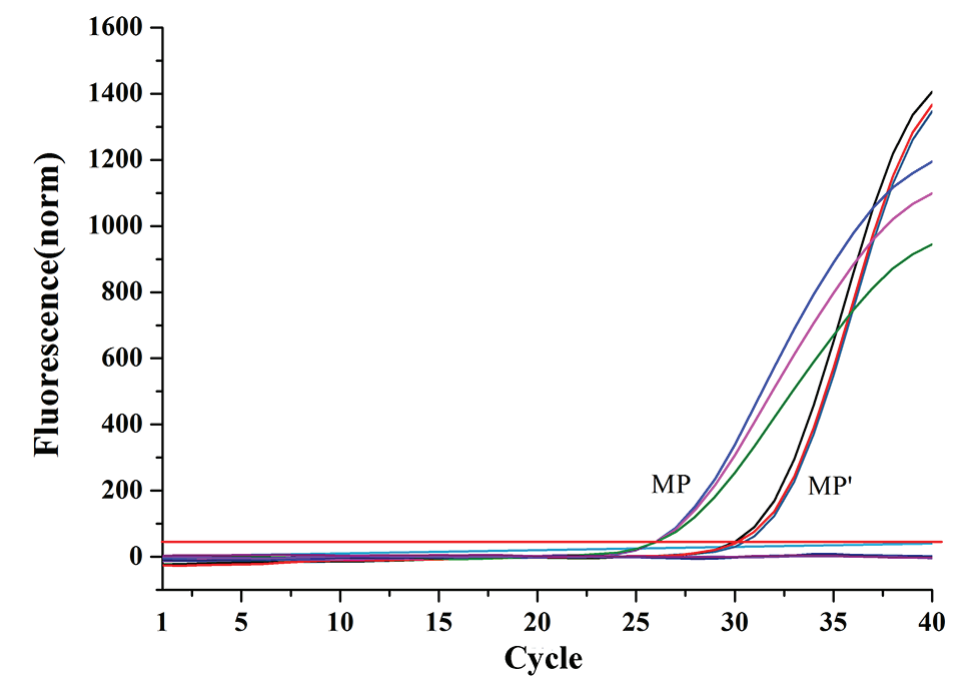
Comparison of the capture capabilities of two magnetic capture probes based on qPCR assays (MP: magnetic capture probe prepared using Method 1; MP′: magnetic capture probe prepared using Method 2).

Twenty micrograms of each of these two magnetic probes were used to capture the same amount of target (1 ml of Long Oligonucleotides1). And then the magnetic pellets were used as DNA templates for qPCR. The results of qPCR (Figure 3) showed that the magnetic capture probes prepared using SMCC had higher separation efficiency (*n* = 3, *p* < 0.05), and combined with the calibration curve (y = −3.1654x + 40.455, *R^2^* = 0.9923) which was established based on Ct values of qPCR using a set of Long Oligonucleotides1solutions of known concentration, the capture capability of MP was 67.4 times than MP′. Therefore, we used the probes based on SMCC-strategy in the following experiments.

### Evaluation of the Amount of Oligonucleotides Immobilized on the Surface of ASMNPs

It is inaccurate to evaluate the number of capture sequences immobilized on the surface of ASMNPs by counting the number of the captured target ssDNA because not all of the capture sequences would hybridize with the target sequences. To obtain more direct data, we immobilized the longer thiol-modified oligonucleotides (Long Oligonucleotides2) on the surface of ASMNPs. We could therefore roughly estimate the amount of immobilized capture sequences by qPCR. Twenty micrograms of magnetic capture probes were used as templates for qPCR resulting in a Ct value of 23.39 (*n* = 3). According to the calibration curve (y = −5.564x + 86.311, *R^2^* = 0.9988) which was obtained based on the serial dilution of Long Oligonucleotides 1, the amount of capture sequences was 10^11.3^ copies per 20 μg of magnetic capture probes. In general, the maximum amount of pathogen in culture media could reach 10^9^ CFU/ml. Therefore, under ideal conditions and regardless of the hybridization rate and the recovery of the magnetic capture probes, even 20 μg of magnetic capture probes would be sufficient to separate the maximum amount of pathogen-derived nucleic acid. In order to further increase the probability of capture, 50 μg was used in the practical application.

### Effects of Magnetic Capture Probes on Polymerase Chain Reaction

**Figure 4:**
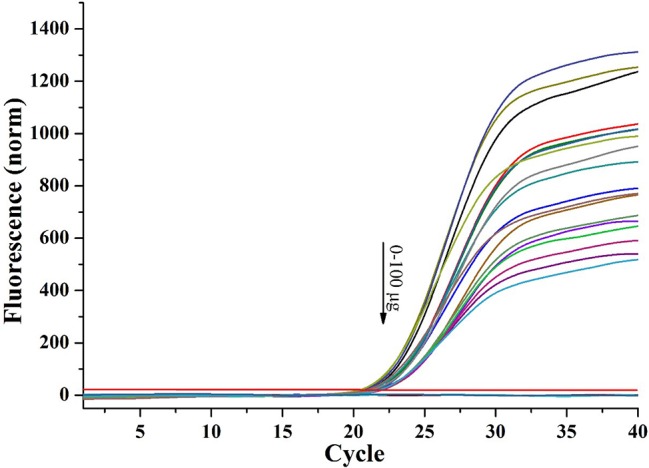
Effect of magnetic capture probe on qPCR (DNA templates: 5 μl of 8.359 ng/μl long oligonucleotides1).

In previous studies, target sequences were always denatured from magnetic capture probes and tediously purified before PCR (Jacobsen and Holben, 2007). In an attempt to optimize sensitivity and detection speed, we planned to directly add the complexes of probes and target sequences in qPCR as templates. However, firstly we needed to identify whether the magnetic capture probes would inhibit qPCR. We varied the amounts to determine the maximum that we could add without inhibiting qPCR. Magnetic capture probes (0, 20, 40, 60, 80, and 100 μg) were added to qPCR mixtures. Though maximum fluorescence decreased with an increase of magnetic capture probes added, probably because the magnetic probes quenched part of fluorescence of SYBR Green, the Ct values were unaffected by the addition of 20, 40, and 60 μg (*n* = 3; *p* > 0.1). At the higher levels (80 and 100 μg), the Ct values increased slightly (*n* = 3, *p* > 0.05; Figure 4). The results showed that when the amount of magnetic capture probes added in qPCR was under 60 μg, the amplification was not affected.

### Effect of pH on Hybridization Rate

**Figure 5:**
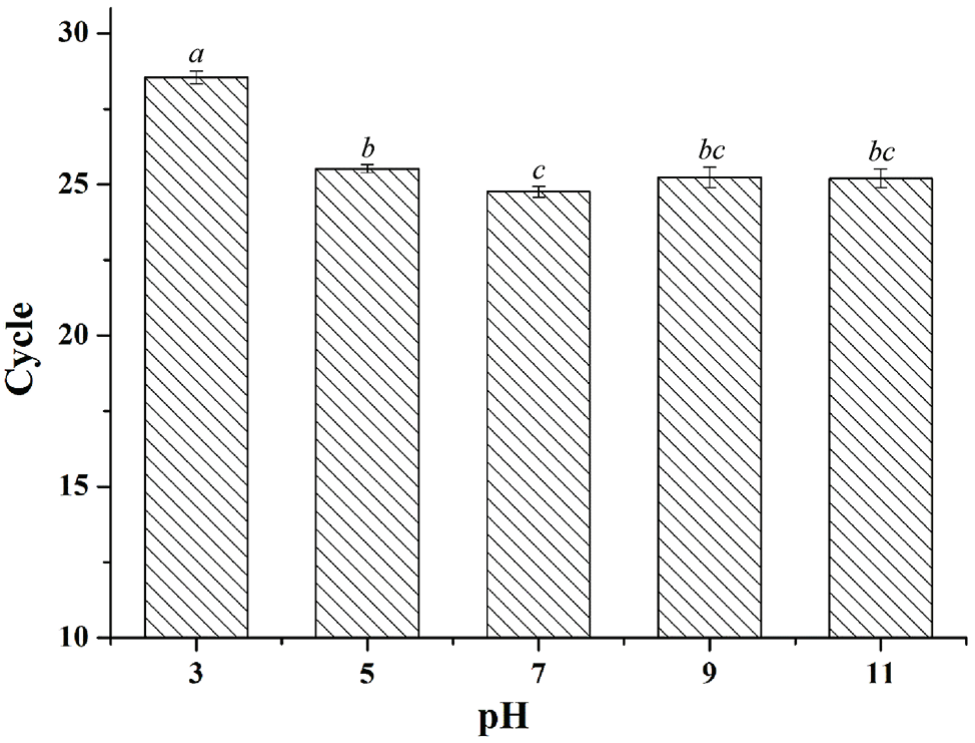
The effect of pH on capture capability of magnetic probes.

The pH of hybridization systems may vary with sample type so we investigated whether pH affected the hybridization rate between the capture sequences and target sequences. Before hybridization, the solutions containing the same amount of Long Oligonucleotides1 were adjusted to different pH with sodium hydroxide and hydrochloric acid. After hybridization and magnetic separation, the pellets were washed with TE buffer and then used as qPCR templates. The hybridization was severely affected only at low pH (pH 3), and there were only slight effects at other levels (Figure 5). That is, even when the solution was treated with Trizol (pH 5), the hybridization would not be much affected.

### Capture Capacity of Magnetic Capture Probes for Long Oligonucleotides1

**Figure 6:**
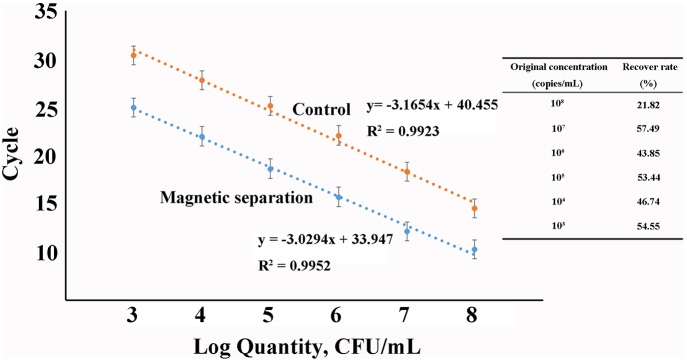
The capture capacity of magnetic probes for artificial long oligonucleotides (Inset: recover rate in solutions with different concentrations).

In order to evaluate the capture capacity of the magnetic capture probes for isolating the target sequences, we used the Long Oligonucleotides1 as a model. These contained sequences for both hybridization capture and qPCR detection. The oligonucleotides were serially diluted 10-fold and 20 μg of magnetic capture probe was hybridized with the targets. All recovery rates were near 50% except for the solution whose original concentration was 10^8^ copies/ml (recovery rate = 22%) (*n* = 3, *p* < 0.05; Figure 6). Although 20 μg of magnetic capture probes might contain more than 10^11.3^ copies of capture sequences based on the previous experiments, they were not sufficient for 10^8^ copies of target sequences. The most probable reason for these results was steric hindrance.

### Detection of *Salmonella* in Milk

**Figure 7:**
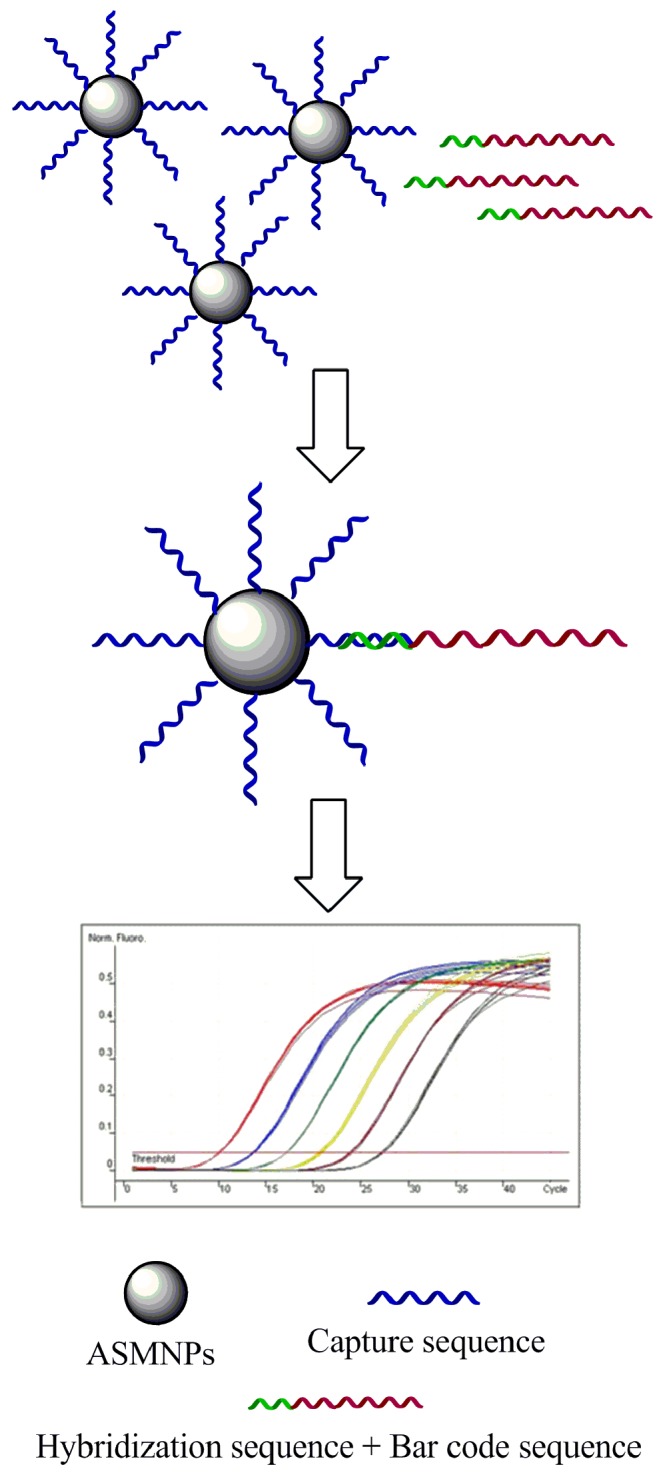
Schematic diagram of the total detection procedure.

**Figure 8:**
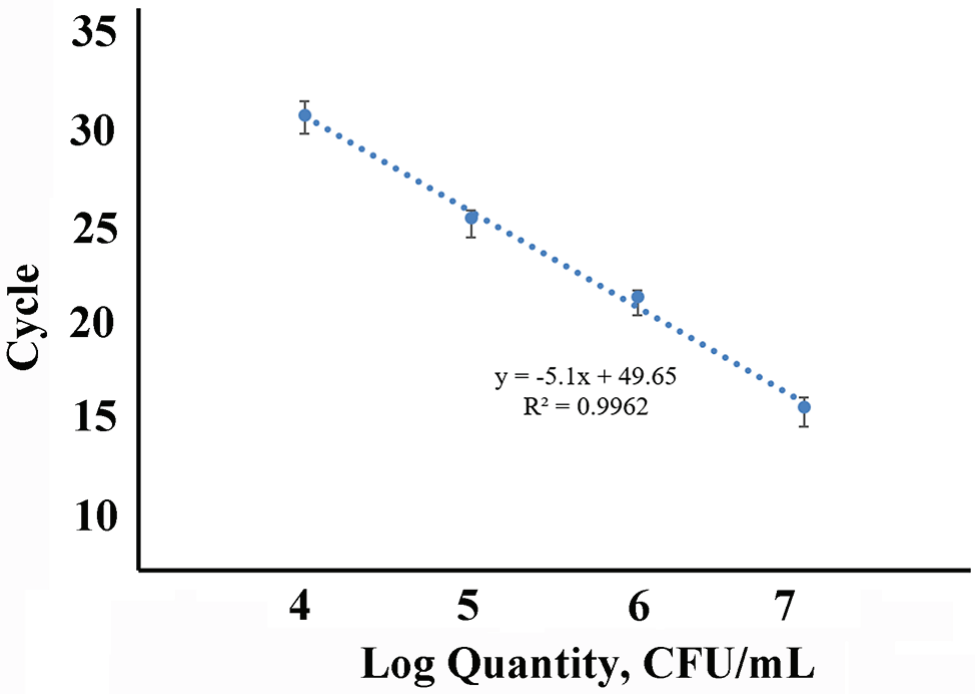
Sensitivity of the detection for *Salmonella* in artificially contaminated milk.

We used magnetic capture probes labeled with Capture Oligonucleotides3, which was partly complementary to *invA* mRNA, to separate the *invA* mRNA in the milk contaminated artificially with 10-fold diluted *Salmonella* and then detected the *invA* mRNA by RT-qPCR. The schematic diagram was shown in Figure 7. The milk was processed according to 2.5; in this case, the target mRNA was released into the solution. When the magnetic capture probes were added, the labeled sequences would hybridize with *invA* mRNA. After magnetic separation and rinse, the magnetic pellets were used to do the RT-qPCR assay. The detection limit was 10^4^ CFU/ml and log-linear relationships occurred from 10^4^ to 10^7^ CFU/ml (Figure 8).

Alternatively, we could enrich the bacteria to get a higher sensitivity. Milk (25 ml) contaminated with *Salmonella* was combined with 225 ml of Luria-Bertani broth and incubated at 37°C for 12 h, then we processed 10 ml of the solution as for the milk samples above. The detection limit reached 10 CFU/ml with a detection rate of 100% (Table 2).

**Table 2:** Detection of *Salmonella* in artificially contaminated milk.

Inoculum (CFU/ml)	Enrichment time (h)				
	0	6	12	18	24
10^−1^	−	−	−	−	−
10^0^	−	−	+	+	+
10^1^	−	−	+	+	+
10^2^	−	−	+	+	+

In addition, 28 bacteria were used to determine the specificity of this method based on extraction of mRNA using magnetic capture probes and RT-qPCR (Table 3). Eighteen *Salmonella* (10^4^ CFU/ml) which was separated from the food samples and identified by our labs were tested, and all showed positive results. Non-*Salmonella* bacteria (10 genera, 10^6^ CFU/ml) showed negative results (no signal before 32 Ct). The results were expected because *invA* gene were proved previously many times to be a specific gene for *Salmonella* (Wang et al., 2015; Pande et al., 2016), and most of all, in this method, the target mRNA were separated firstly by using magnetic probes; thus, the specificity would be better than those methods in which all of the mRNA was used to convert into cDNA.

**Table 3:** Specificity of the method based on extraction of mRNA using magnetic capture probes and RT-qPCR.

*Salmonella* strains			Non-*Salmonella* strains		
Organism	Number	Result	Organism	Number	Result
*Salmonella typhimurium*	5	+	*Staphylococcus aureus*	3	
*Salmonella choleraesuis*	4	+	*Listeria monocytogenes*	2	−
*Salmonella enteritidis*	3	+	*Vibrio parahaemolyticus*	1	−
*Salmonella paratyphi*	2	+	*Escherichia coli*	1	−
*Salmonella infantis*	1	+	*Shigella dysenteriae*	1	−
*Salmonella tallahassee*	1	+	*Bacillus subtilis*	1	−
*Salmonella vellore*	1	+	*Enterococcus faecium*	1	−
*Salmonella anatum*	1	+			

## Discussion

*Salmonella* is the leading cause of bacterial food poisoning in humans worldwide (Zhang et al., 2016; Rahman, 2017). It is reported that more than 90% of human illness caused by *Salmonella* is foodborne and results from contaminated meat, eggs, and milk (Foley and Lynne, 2008). Thus, more rapid and reliable methods for the detection of *Salmonella* are required except the traditional culture methods which take 4–7 days. For culture-independent methods, magnetic separation has been widely used to enrich the targets to realize rapid detection (Brandão et al., 2015). In our previous research, we found ASMNPs could adsorb DNA by hydrogen bond and electrostatic interaction, and thus, we used ASMNPs to separate bacterial genomic DNA and combined it with PCR to rapidly detect *Salmonella* Enteritidis *and L. monocytogenes* (Bai et al., 2013). However, pathogen detection *via* DNA does not differentiate between viable and dead bacteria because DNA from non-viable bacteria also could produce signal. Since mRNA is very labile, it is considered that detection of mRNA is superior to detection based on DNA. Therefore, in this study, we prepared magnetic capture probes to separate mRNA sequences by hybridization and using RT-qPCR in which complexes of probes and target sequences were directly used as template to detect *Salmonella*.

For improving the separation capability of the magnetic capture probes, on the one hand, we prepared the magnetic nanoparticles with rich amino groups based on previous research (Bai et al., 2016); on the other hand, we selected a superior method, SSMC-based strategy, to couple the oligonucleotides with amino groups to make sure the probes contain more capture sequences.

Further, to reduce the operating steps, save time, and improve sensitivity, we tried to directly add the complexes of magnetic capture probes and target sequences to PCR. In previous research, we found that the bare ASMNPs would inhibit PCR by adsorbing PCR components and the amplification would be normal after the surface of ASMNPs were blocked. The magnetic capture probes were also blocked by capture sequences and proteins; thus, appropriate amount of probes could not affect PCR amplification. Moreover, the magnetic capture probes separated mRNA sequences would work in the solution containing Trizol (pH 5.0) in order to simplify the mRNA isolation steps. In this case, the effect of pH on the hybridization rate should be evaluated. After comparing different pH conditions, we found there was only slight effect for hybridization at pH 5.

The magnetic capture probes were used to isolate the *invA* mRNA in milk artificially contaminated with *Salmonella*, and the *invA* mRNA was then detected by RT-qPCR. The detection limit was 10^4^ CFU/ml (about 30 Ct). At the same time, we noted that a Ct value (about 32) existed with no *Salmonella* added to the milk. Though we could confirm this was caused by the magnetic capture probes, the detailed reason is still not clear and needed to be explored in future research. That is, if we clearly know that why the magnetic capture probes caused a false positive signal at 32 cycles in RT-qPCR without target mRNA, maybe we could realize a lower detection limits. But even so, the current detection limit was superior. Fey et al. reported that the detection limits were 5 × 10^4^ and 5.5 × 10^4^ copies (*invA* gene) in drinking and pond water, respectively (Fey et al., 2004). In this research, the sample was milk which was more complex than water. Most of all, this method was more rapid and simple to extract target mRNA. Alternatively, after cultivation for 12 h, the detection rate was 100% even though the milk contained only 10 CFU/ml of *Salmonella*. This result was also superior to our previous research extracting mRNA based on the traditional method. In our previous research (Zhou et al., 2014), the samples must be cultivated for 18 h to detect 10 CFU/ml of *Salmonella*.

## Author Contributions

YB designed and initiated the study, interpreted the results, and wrote the manuscript. YC and XS contributed to improvement of the manuscript. YS, CS, and DW contributed to analysis of the data and discussion of the results. XS designed the outline of this study and manuscript and provided laboratory equipment and space.

### Conflict of Interest Statement

The authors declare that the research was conducted in the absence of any commercial or financial relationships that could be construed as a potential conflict of interest.
